# Correction to “Non‐Small Cell Lung Cancer Patients With Tumors ≤ 2 cm Are Suitable for Wedge Resection or Segmentectomy: A Real‐World Study”

**DOI:** 10.1111/1759-7714.70303

**Published:** 2026-05-27

In the originally published version of the above article, there was a data entry error in the abstract, Figures 2–4, Tables 3, 7, and 8, and the corresponding data descriptions in the main text. Specifically, the data for OS and LCSS were inadvertently swapped during table preparation.

The positions that should have presented OS data were incorrectly filled with LCSS data. The positions that should have presented LCSS data were incorrectly filled with OS data.

The author reviewed the original data and corrected the tables and related text. All OS and LCSS data, as well as their corresponding figures in the text, have now been placed in their respective correct positions as detailed below:

**Abstract (Results)**

A total of 640 patients were enrolled (wedge resection: 295; segmentectomy: 345). After IPTW, no difference in baseline characteristics was observed between the two groups. Additionally, long‐term outcomes did not significantly differ between the groups. However, compared with segmentectomy, wedge resection was associated with a shorter operation duration (*p* < 0.001), less intraoperative blood loss (*p* < 0.001), fewer complications (*p* < 0.001), and shorter postoperative stay (*p* = 0.047). In the subgroup with a consolidation‐to‐tumor ratio (CTR) > 0.25, segmentectomy resulted in longer OS (*p* = 0.036), LCSS (*p* = 0.046) as well as higher 5‐year OS (*p* = 0.015), 5‐year RFS (*p* = 0.023), and 5‐year LCSS (*p* = 0.045).

**3.3.1 Comparison of Long‐Term Survival Outcomes**

A total of 640 patients completed long‐term follow‐up. The median follow‐up was 86.5 months for the wedge resection group and 88.4 months for the segmentectomy group. The 5‐year OS rates were 94.91% for the wedge resection group and 96.52% for the segmentectomy group (*p* = 0.314). The 5‐year RFS rates were 93.89% versus 93.04% (*p* = 0.663), and the 5‐year LCSS rates were 96.61% versus 97.39% (*p* = 0.562) for the wedge resection and segmentectomy groups, respectively. Similarly, after IPTW adjustment, no significant differences in survival outcomes were observed between the two groups: OS (*p* = 0.790), RFS (*p* = 0.982), LCSS (*p* = 0.759), 5‐year OS (*p* = 0.129), 5‐year RFS (*p* = 1.000), and 5‐year LCSS (*p* = 0.244). The long‐term outcomes are summarized in Table 3. The Kaplan–Meier survival curves for OS, RFS, and LCSS are shown in Figure 2.

**3.4.2 Subgroup With a CTR > 0.25**

Patients with NSCLC with a CTR > 0.25 were selected for subgroup analysis. The total sample size before matching was 89 patients, including 42 who underwent wedge resection and 47 who underwent segmentectomy. The results of the balance analysis before matching revealed that compared with the wedge resection group, the segmentectomy group had significantly longer OS (*p* = 0.005), RFS (*p* = 0.045), LCSS (*p* = 0.010), 5‐year OS (*p* = 0.002), 5‐year RFS (*p* = 0.005), and 5‐year LCSS (*p* = 0.001). The results of the balance analysis after matching revealed that compared with the wedge resection group, the segmentectomy group had significantly longer OS (*p* = 0.036), RFS (*p* = 0.061), LCSS (*p* = 0.046), 5‐year OS (*p* = 0.015), 5‐year RFS (*p* = 0.023), and 5‐year LCSS (*p* = 0.045), as shown in Table 8. The Kaplan–Meier curves for OS, RFS, and LCSS in the wedge resection and segmentectomy groups for patients with a CTR > 0.25 are shown in Figure 4.

The corrected Figures 2, 3, 4 and Tables 3, 7, and 8 are presented below.

**FIGURE 2:**
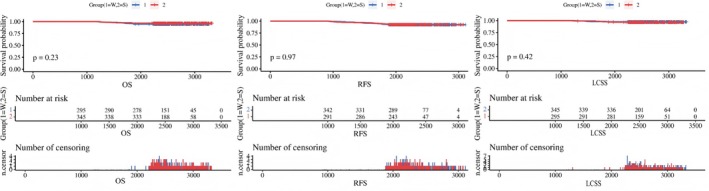
Kaplan Meier survival curves of OS, RFS, LCSS.

**FIGURE 3:**
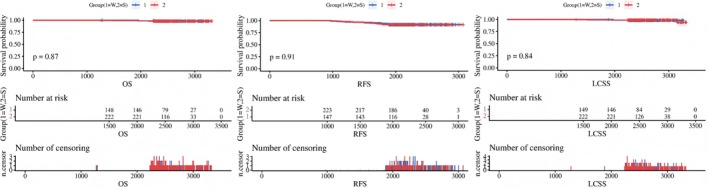
Kaplan Meier survival curves of OS, RFS, LCSS in CTR > 0 subgroup.

**FIGURE 4:**
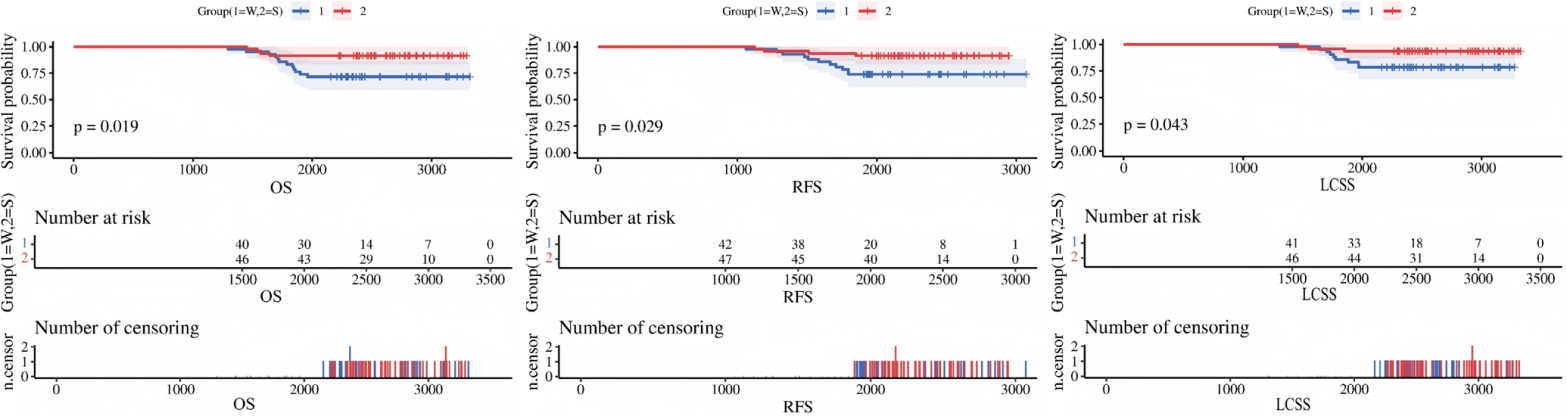
Kaplan Meier survival curves of OS, RFS, LCSS in CTR > 0.25 subgroup.

**TABLE 3:** Long‐term outcomes between two groups.

	Entire cohort			IPTW cohort		
	Wedge resection (*n* = 295)	Segmentectomy (*n* = 345)	*p* value	Wedge resection (*n* = 284)	Segmentectomy (*n* = 284)	*p* value
OS(day), Mean±SD	2566.458±378.461	2605.304±372.517	0.192	2621.259±385.789	2635.553±308.506	0.790
RFS(day), Mean±SD	2229.529±344.404	2242.217±359.313	0.650	2270.635±339.126	2269.494±334.035	0.982
LCSS(day), Mean±SD	2591.047±365.830	2626.299±361.685	0.222	2633.224±384.368	2649.576±305.460	0.759
5‐year OS, *n* (%)	280 (94.915)	333 (96.522)	0.314	281 (98.597)	285 (100.000)	0.129
5‐year RFS, *n* (%)	277 (93.898)	321 (93.043)	0.663	282 (98.947)	281 (95.294)	1.000
5‐year LCSS, *n* (%)	285 (96.610)	336 (97.391)	0.562	282 (98.947)	285 (100.000)	0.244

**TABLE 7:** Survival data analysis results of subgroup with CTR > 0.

	Entire cohort			IPTW cohort		
	Wedge resection (*n* = 149)	Segmentectomy (*n* = 225)	*p* value	Wedge resection (*n* = 219)	Segmentectomy (*n* = 219)	*p* value
OS(day), Mean±SD	2578.034±405.474	2558.707±370.981	0.635	2604.139±362.617	2617.715±363.678	0.757
RFS(day), Mean±SD	2227.651±368.130	2210.169±342.721	0.639	2253.109±325.424	2265.854±325.866	0.746
LCSS(day), Mean±SD	2601.758±382.264	2591.360±360.895	0.790	2621.737±347.509	2635.007±366.824	0.759
5‐year OS, *n* (%)	139 (93.289)	214 (95.111)	0.454	212 (97.080)	212 (97.080)	1.000
5‐year RFS, *n* (%)	139 (93.289)	208 (92.444)	0.757	214 (97.810)	212 (97.080)	1.000
5‐year LCSS, *n* (%)	141 (94.631)	219 (97.333)	0.178	214 (97.810)	212 (97.080)	1.000

**TABLE 8:** Survival data analysis results of subgroup with CTR > 0.25.

	Entire cohort			IPTW cohort		
	Wedge resection (*n* = 42)	Segmentectomy (*n* = 47)	*p* value	Wedge resection (*n* = 41)	Segmentectomy (*n* = 41)	*p* value
OS(day), Mean±SD	2346.000±556.668	2647.532±406.288	0.005	2283.154±367.610	2575.846±399.574	0.036
RFS(day), Mean±SD	2116.524±525.903	2306.681±311.624	0.045	2123.154±383.548	2311.692±411.173	0.061
LCSS(day), Mean±SD	2443.738±535.443	2700.915±381.611	0.010	2303.731±377.729	2615.923±406.655	0.046
5‐year OS, *n* (%)	30 (71.429)	45 (95.745)	0.002	32 (78.049)	37 (90.244)	0.015
5‐year RFS, *n* (%)	30 (71.429)	44 (93.617)	0.005	31 (75.610)	36 (87.805)	0.023
5‐year LCSS, *n* (%)	31 (73.810)	46 (97.872)	0.001	37 (90.244)	40 (97.561)	0.045

There were no scientific conclusions of the article that remain unchanged.

We apologize for this error.
